# Cross-sectional analysis of the association between physical activity and metabolic syndrome in the elderly

**DOI:** 10.3389/fendo.2026.1799367

**Published:** 2026-03-25

**Authors:** Chuandi Zhang, Chunxia Shen, Xiaoyan Yu, Chunjie Ou, Tiane Liu

**Affiliations:** 1 Department of Public Health, Hangzhou Linping District Hospital of Integrated Traditional Chinese and Western Medicine, Hangzhou, Zhejiang, China; 2 Department of Expanded Program on Immunization, Hangzhou Linping District Center for Disease Control and Prevention (Hangzhou Linping District Health Supervision Institute), Hangzhou, Zhejiang, China

**Keywords:** body mass index, cross-sectional analysis, elderly, metabolic syndrome, physical activity

## Abstract

**Background:**

Metabolic syndrome (MetS) is a critical cluster of risk factors for cardiovascular and cerebrovascular diseases. Exploring effective prevention and management strategies for MetS is of great significance for promoting healthy aging. Although physical activity (PA) is widely recommended for MetS prevention, the association between PA levels and MetS, as well as its diagnostic components, in the elderly population remains insufficiently studied.

**Methods:**

A cross-sectional study design was employed, involving 1,651 permanent residents aged ≥60 years who participated in a community health examination in Linping District from June to July 2024. PA levels, weekly activity duration, and total activity volume were assessed using the International Physical Activity Questionnaire (IPAQ). MetS and its components were diagnosed according to the International Diabetes Federation (IDF) criteria. Multivariate logistic regression was used to analyze the association between PA and MetS and its components, with sensitivity analyses and subgroup analyses stratified by body mass index (BMI) performed.

**Results:**

The prevalence of MetS in the study population was 49.12%. Multivariate regression analysis showed no significant association between PA levels and overall MetS risk after adjusting for confounding factors (*P* > 0.05). Increased weekly activity duration was weakly positively associated with MetS risk (*OR* = 1.01, *95% CI*: 1.01~1.01, *P* = 0.025). Moderate PA level (*OR* = 1.35, *95% CI*: 1.05~1.73), weekly activity duration (*OR* = 1.01), and total activity volume (*OR* = 1.00) were all positively associated with elevated triglyceride (TG) risk (all *P* < 0.05). Subgroup analysis revealed a significant interaction with BMI (*P* for interaction = 0.006): moderate-to-vigorous physical activity (MVPA) was positively associated with elevated TG risk only in the normal-weight group (*OR* = 1.71, *95% CI*: 1.25~2.35, *P* < 0.001), with no such association observed in underweight, overweight, or obese groups.

**Conclusion:**

In this elderly population, no expected inverse association was found between overall PA levels and MetS risk. Instead, longer activity duration and moderate PA were associated with an increased risk of elevated TG, particularly among normal-weight elderly individuals. These findings suggest that metabolic health management for the elderly should adopt an integrated strategy combining PA, weight control, and individualized assessment.

## 1 Introduction

With social progress, economic development, advancements in medical technology, and improvements in public health conditions, global life expectancy continues to increase, and the aging process continues to accelerate. The health issues of the elderly population have increasingly become a focus in the field of public health (1). As of 2019, 88 countries worldwide have been defined as aging societies (2). The elderly population generally exhibits epidemiological characteristics of high disease prevalence, accompanied by age-related pathophysiological specificities such as muscle decline, enhanced inflammatory status, and changes in hormone levels. These factors significantly increase the risks of cardiovascular and cerebrovascular events, cognitive decline, and all-cause mortality. At the same time, the duration of living with chronic conditions among the elderly is gradually lengthening, which not only increases the individual burden of disease but also places sustained pressure on public healthcare systems (3).

Metabolic syndrome (MetS) is a cluster of clinical conditions including central obesity, abnormal blood glucose, hypertension, and dyslipidemia. It serves as a significant cluster of risk factors for cardiovascular diseases and type 2 diabetes (4). Its characteristics include obesity, insulin resistance, metabolic inflammation, endothelial dysfunction, pro-thrombotic states, atherosclerosis, and impaired host defense functions (5). The prevalence of MetS continues to rise. Initially emerging in high-income countries driven by unhealthy behaviors such as lack of physical activity and poor diet, it has now developed into a global epidemic (6). MetS is a key risk factor leading to functional decline and reduced quality of life among the elderly, significantly increasing all-cause mortality, disability rates, and healthcare burdens in this population (7). According to the World Health Organization (WHO), between 2006 and 2015, deaths caused solely by stroke, MetS, diabetes, and heart disease resulted in a loss of $558 billion in national income in China (8). Therefore, exploring effective prevention and management strategies for MetS is of great significance for promoting healthy aging.

Physical activity (PA) refers to all activities that increase the body’s energy expenditure through skeletal muscle contractions, encompassing various aspects such as daily life, household chores, leisure activities, physical exercise, and daily commuting (9). Various forms of PA, including leisure, occupational, transportation, and household activities, as well as aerobic, resistance, flexibility, and comprehensive training, all contribute to optimizing physical function, enhancing muscle strength, reducing functional impairment, and delaying the progression of chronic diseases (10, 11). Numerous studies have confirmed that PA offers multifaceted health benefits, including promoting physical and mental health, slowing the aging process, reducing the risk of disability, and improving quality of life. It effectively lowers the risk of chronic diseases such as hypertension, diabetes, cardiovascular diseases, and cancer (12–14), reduces the risk of premature death (15), and significantly promotes healthy aging. PA has been proven to positively reduce the risk of MetS and is recommended as an effective treatment for MetS. A systematic review and meta-analysis focusing on elderly individuals with type 2 diabetes indicated that aerobic exercise appears to be the most effective form of PA for improving MetS, particularly when performed over longer durations (16). A 12-week study showed that remote training programs effectively increased physical activity levels and cardiorespiratory endurance in patients with multiple cardiometabolic diseases (17). A study on metabolic syndrome and moderate-to-vigorous physical activity (MVPA) among Asian American adults found that those not meeting the recommended standard (≥150 minutes per week) had a 1.5 times higher likelihood of developing MetS compared to those who met the standard (18). Moderate PA can improve body composition, enhance cardiorespiratory fitness, and positively regulate various biochemical indicators, such as lowering fasting blood glucose, modulating lipid metabolism, stabilizing blood pressure, and exerting anti-inflammatory effects (19–22). These pathways are central to combating the various components of MetS. Therefore, increasing PA in daily life is considered crucial for preventing MetS and holds significant value in reducing the incidence and mortality of chronic diseases, as well as decreasing healthcare costs and losses in social productivity.

However, with technological advancements and economic development, reliance on convenient lifestyles such as motorized transportation and food delivery services has become increasingly common, leading to insufficient PA and exacerbating the occurrence of chronic diseases like diabetes, dyslipidemia, and obesity (23). The Global Burden of Disease Study on behavioral and metabolic risk factors showed that between 2006 and 2016, disability-adjusted life years attributable to insufficient PA increased by 15.4% (24). Due to the natural decline in physiological function with age, physical activity capacity, balance, and walking ability often diminish in the elderly. This makes it challenging for older adults to engage in normal PA, leading them to adopt more sedentary lifestyles (25, 26). Based on this, the WHO and various national health guidelines strongly recommend that adults and the elderly engage in regular and sufficient PA to maintain physical function and prevent diseases (27–29). The American College of Sports Medicine and the American Heart Association, in their Recommendations for Physical Activity and Public Health, advise that adults should engage in at least 150 minutes of moderate-intensity aerobic activity per week, or 75 minutes of vigorous-intensity PA per week, or an equivalent combination, to reduce all-cause, cardiovascular disease, and cancer mortality (30). The WHO, in its guidelines on physical activity and sedentary behavior, states that even elderly individuals with chronic diseases or disabilities should engage in diversified physical activities, including at least moderate- or high-intensity balance and strength training on three or more days per week (27). Personalized PA programs, tailored to individual goals and preferences, can effectively improve adherence among the elderly (31).

A meta-analysis of elderly type 2 diabetes patients showed that aerobic exercise can effectively improve various components of metabolic syndrome (MetS) (16). However, this study and many other related studies often rely on a single physical activity (PA) assessment metric, such as whether the weekly activity time threshold is met (18), which may not fully reflect the complete characteristics of activity patterns. Additionally, existing research evidence mostly comes from middle-aged and young populations (32, 33), while the physical activity patterns, intensity, types, and health status of the elderly population are unique. The metabolic benefits they gain from physical activity may differ from those of younger groups, and they may even require higher levels of activity to achieve similar effects (34). Meanwhile, large epidemiological studies commonly rely on self-reported measurement methods, which remains a common limitation and may introduce recall bias. Notably, previous studies have generally not identified or deeply explored heterogeneous associations between different dimensions of physical activity (such as activity level, duration, and total volume) and individual components of metabolic syndrome, nor have they fully clarified the moderating effects of factors like BMI on these associations in the elderly population.

Based on the aforementioned research gaps, this study systematically analyzed, within a large community-based sample of Chinese older adults, the independent associations between multiple dimensions of PA—namely, intensity level, weekly duration, and total metabolic equivalents—and both MetS and each of its five components. We further explicitly explored the role of BMI as a potential effect modifier in these associations, an approach that allows for hypothesis generation regarding potentially vulnerable subgroups, aiming to provide more refined epidemiological evidence for developing targeted and personalized PA recommendations for the elderly.

The primary aim of this study was to investigate the cross-sectional associations between multidimensional PA metrics and MetS, as well as its individual components, in a community-dwelling elderly population. We hypothesized that higher PA levels, duration, and volume would be inversely associated with MetS prevalence, but that these associations might vary across different MetS components and be modified by factors such as BMI.

## 2 Materials and methods

### 2.1 Study design and participants

This study utilized cross-sectional data from the annual free health examination program for community-dwelling elderly residents, organized by the Linping District Health Bureau. All participants were permanent residents who took part in the examination at their local community health service center between June 1st and July 31st, 2024. The health examination is a standard public health service provided to all registered residents aged 60 and above.

Inclusion criteria were as follows: a) Age ≥ 60 years. b) Residence in the community for ≥ 5 years, defined as having a registered household address and having lived primarily within the catchment area of the community health service center for a minimum of five consecutive years, as verified by the local residential registration system. c) Clear consciousness and ability to cooperate with the survey. Exclusion criteria included: a) History of severe heart, liver, or kidney insufficiency or malignant tumors, were truthfully recorded if they answered “yes” to any of these conditions based on self-reported physician diagnoses during face-to-face interviews conducted by trained community health workers prior to the physical examination. b) History of severe mental illness or cognitive impairment, assessed using the same self-report physician diagnostic method in individuals. c) Inability to perform daily PA due to reasons such as physical disability. d) Missing data for key variables.

A total of 1,823 elderly individuals were initially enrolled in this study for health examination registration. After applying exclusion criteria, 172 participants were excluded due to missing data in the PA questionnaire. A total of 1651 elderly individuals were finally included in the analysis. Based on the presence or absence of MetS, participants were divided into a MetS group (n=811) and a non-MetS group (n=840). A complete-case analysis approach was therefore used, as the proportion of missing data was low (9.43%) and unlikely to introduce substantial bias.

### 2.2 Data collection

Collected data included: demographics, lifestyle, physical activity assessment, physical measurements, and blood sample testing.

a) Socio-demographic data: Age, gender.
b) Lifestyle: Smoking status (defined as “current smoker” or “non-smoker/former smoker”), alcohol consumption status (defined as “current drinker” or “non-drinker/former drinker”). Smoking status was dichotomized as “current smoker” vs. “non-smoker/former smoker” to capture the effect of active smoking on metabolic health. Drinking history was categorized based on weekly frequency into occasional drinking (≤3 times/week), frequent drinking (3–7 times/week), daily drinking (≥7 times/week), and former drinker, although it was adjusted as a binary variable (current vs. non/former drinker) in the main regression models for parsimony, consistent with common practice in metabolic syndrome research.
c) Physical activity assessment:

The International Physical Activity Questionnaire (IPAQ) (35) was used to assess participants’ PA related to work, transportation, household/gardening, and leisure over the past week, recording PA type, weekly frequency, daily duration, and years of activity. IPAQ classifies PA intensity into three levels: vigorous (e.g., heavy lifting, fast cycling, aerobic exercise), moderate (e.g., normal-speed cycling, ball games, light lifting), and low (e.g., walking). Weekly activity duration: Total minutes per week for all types of PA (walking, moderate, vigorous). Weekly Activity Volume: Total metabolic equivalent (MET-min/week) for all types of PA.

PA intensity categorization was based on Metabolic Equivalent (MET) conversion. The MET value for each activity was multiplied by its weekly duration and summed to calculate total activity volume (MET-min/week). According to IPAQ criteria, PA intensity was classified into three groups: high, moderate, and low (36). The cutoff points for these categories were based on the standard metabolic equivalent, which combines the frequency, duration, and intensity of activity to reflect the public health guidelines for promoting adequate PA for health. The aim was to classify individuals into activity levels with known health impacts, ensuring that our findings could be compared with studies extensively utilizing the IPAQ. High Activity Group: Meeting either of the following two criteria: ① Total days of vigorous PA ≥ 3 AND total weekly PA ≥ 1500 MET-min/week. OR ② Total days of all three intensities combined ≥ 7 AND total weekly PA ≥ 3000 MET-min/week. Moderate Activity Group: Meeting any of the following three criteria: ① At least 20 minutes of vigorous PA per day, on ≥ 3 days/week. OR ② At least 30 minutes of moderate PA and/or walking per day, on ≥ 5 days/week. OR ③ Total days of all three intensities combined ≥ 5 AND total weekly PA ≥ 600 MET-min/week. Low Activity Group: Meeting either of the following two criteria: ① No activity reported. OR ② Some activity reported but not meeting the above moderate or high group criteria.

The IPAQ assesses PA across four domains: work, transportation, household/gardening, and leisure-time. For example, participants reported time spent on activities such as walking for transportation, heavy lifting at work (vigorous), cycling at a normal pace (moderate), and light household chores like sweeping (moderate). This provides a comprehensive, though self-reported, picture of daily life activities.

d) Physical measurements:

Professional medical staff performed standardized measurements of height, weight, waist circumference, and blood pressure. ① Height and Weight: Participants wore light clothing and removed shoes. Body Mass Index (BMI) was calculated as: BMI = weight (kg)/[height (m)]². ② Waist Circumference: Measured at the midpoint between the lower border of the 12th rib and the iliac crest. ③ Blood Pressure: After resting for 5 minutes, blood pressure was measured three times on the right upper arm, and the average was taken as systolic (SBP) and diastolic (DBP) blood pressure.

All physical measurements were conducted by trained community health workers and nurses who underwent a standardized training session prior to data collection. This training covered the correct use of all equipment and standardized measurement protocols. Height and weight were measured using an automatically calibrated stadiometer and scale. Blood pressure was measured using an automated sphygmomanometer, which was calibrated daily.

e) Blood testing:

All participants fasted for 8–12 hours, and 5 mL of venous blood was collected in the morning. Serum was separated by centrifugation and stored at -20 °C until analysis. Standard biochemical analyzers were used to measure: Triglycerides (TG), High-Density Lipoprotein Cholesterol (HDL-C), Fasting Plasma Glucose (FPG).

### 2.3 Definition of variables

a) MetS: Diagnosed according to the International Diabetes Federation (IDF) criteria for Asian populations (37): Central obesity (male waist circumference ≥90 cm, female waist circumference ≥80 cm) plus any two of the following four criteria: ① Elevated TG: TG ≥1.7 mmol/L, or on relevant treatment; ② Reduced HDL-C: male <1.03 mmol/L, female <1.29 mmol/L, or on relevant treatment; ③ Elevated BP: SBP ≥130 mmHg or DBP ≥85 mmHg, or on antihypertensive treatment or previously diagnosed hypertension; ④ Elevated FPG: FPG ≥5.6 mmol/L, or previously diagnosed type 2 diabetes or on relevant treatment.
b) MetS Components: Based on the above criteria, five binary outcome variables were defined: “Central obesity,” “Elevated BP,” “Elevated TG,” and “Reduced HDL-C,” “Elevated FPG.”
c) BMI: Classified according to WHO standards for Asian adults (38): ① Underweight = BMI <18.5 kg/m²; ② Normal weight = BMI 18.5–24.9 kg/m²; ③ Overweight = BMI 25.0–29.9 kg/m²; ④ Obese = BMI ≥30.0 kg/m².

### 2.4 Statistical analysis

a) R software (version 4.3.0) was used for statistical analysis. Normally distributed continuous data are presented as mean ± standard deviation (Mean ± SD), with comparisons between groups performed using analysis of *t*-test. Non-normally distributed continuous data are presented as median and interquartile range [M (Q_1_, Q_3_)], with comparisons between groups performed using the Mann-Whitney U test. Categorical data are presented as counts and percentages [n (%)], with comparisons between groups performed using the chi-square test.
b) Logistic regression models were used to analyze the association between PA (independent variable) and the risk of MetS (dependent variable) and its components. The independent variable (PA) was incorporated into the models in three ways: ① PA level (with “low level” as reference, using “moderate level” and “high level” as dummy variables); ② Weekly activity duration (as a continuous variable); ③ Weekly total activity volume (MET-min/week, as a continuous variable). Covariates for adjustment in the multivariate logistic regression models were selected based on *a priori* knowledge of their established or potential roles as confounders in the association between PA and MetS. These included: gender and age (basic demographic factors influencing both PA and MetS), BMI (a key mediator and confounder strongly associated with both exposure and outcome), smoking, and alcohol consumption (important lifestyle factors that are known to influence metabolic health and may be associated with PA levels). All models were adjusted for the following covariates: gender, age (continuous), BMI (continuous), smoking (binary), and alcohol consumption (binary). Odds ratios (*OR*) and their 95% confidence intervals (*CI*) were calculated. To minimize information bias, standardized questionnaires and measurement protocols were used, and data collectors were uniformly trained as described above. To reduce confounding bias, we adjusted for a comprehensive set of *a priori* confounders in our multivariate logistic regression models.
c) To test the robustness of the main analysis results, two sensitivity analyses were performed: ① Combining moderate and high PA levels into one group and comparing it with the low-level group; ② Constructing regression models without adjusting for BMI.
d) To explore whether BMI modifies the association between PA and outcomes, stratified analyses were performed by BMI category (underweight, normal weight, overweight, obese). In each stratum, the combined moderate/high PA group was compared with the low PA group, adjusting for gender, age, smoking, and alcohol consumption. The likelihood ratio test was used to compare the goodness-of-fit between models with and without an interaction term (PA group × BMI category), and the P-value for interaction was calculated.

The sample for this analysis was derived from all eligible elderly participants who attended the community health examination during the specified two-month period. This constitutes a convenience sample based on the available data from the public health examination program. However, the final sample of 1,651 participants provides sufficient statistical power (> 0.80) to detect small to moderate effect sizes (odds ratios of approximately 1.3 or higher) for the main associations of interest, assuming a two-sided alpha of 0.05 and the observed prevalence of MetS and PA exposures.

A *P*-value < 0.05 was considered statistically significant for between-group comparisons.

## 3 Results

### 3.1 Basic characteristics of the study population

A total of 1651 elderly individuals aged 60 years and above were included in this study, comprising 811 participants (49.12%) in the MetS group and 840 participants (50.88%) in the non-MetS group. The baseline characteristics of the two groups are shown in **Table 1**.

**Table 1:** Baseline characteristics of the study population by metabolic syndrome status.

Variables	Total (n = 1651)	Non-MetS (n=840)	MetS (n=811)	Statistic	*P*-value
Demographics, n(%)/Mean ± SD					
Gender				*χ*²=209.06	**<.001**
Male	781 (47.30)	544 (64.76)	237 (29.22)		
Female	870 (52.70)	296 (35.24)	574 (70.78)		
Age (years)	68.91 ± 6.84	68.40 ± 7.03	69.43 ± 6.62	*t* = -3.06	**0.002**
Age Group (year)				*χ*²=17.40	**<.001**
60-	524 (31.74)	305 (36.31)	219 (27.00)		
65-	399 (24.17)	197 (23.45)	202 (24.91)		
70-	356 (21.56)	166 (19.76)	190 (23.43)		
75-	372 (22.53)	172 (20.48)	200 (24.66)		
Lifestyle, n(%)					
Smoking				*χ*²=63.35	**<.001**
No	1303 (78.92)	597 (71.07)	706 (87.05)		
Yes	348 (21.08)	243 (28.93)	105 (12.95)		
Drinking				*χ*²=22.68	**<.001**
No	1367 (82.80)	659 (78.45)	708 (87.30)		
Yes	284 (17.20)	181 (21.55)	103 (12.70)		
Physical activity, n(%)/M (Q_1_, Q_3_					
PA Level				χ²=2.91	0.234
Low	1319 (79.89)	662 (78.81)	657 (81.01)		
Moderate	321 (19.44)	170 (20.24)	151 (18.62)		
High	11 (0.67)	8 (0.95)	3 (0.37)		
Activity Time (min/w)	0.00 (0.00, 30.00)	0.00 (0.00, 30.00)	0.00 (0.00, 30.00)	*Z* = -0.95	0.341
Activity Amount (MET-min/w)	0.00 (0.00, 315.00)	0.00 (0.00, 315.00)	0.00 (0.00, 270.00)	Z=-0.64	0.521
Anthropometric & Metabolic indicators, Mean ± SD					
Bmi(kg/m²)	24.32 ± 3.00	22.70 ± 2.44	26.01 ± 2.57	*t* = -26.88	**<.001**
Waist Circumference (cm)	85.32 ± 8.36	80.42 ± 6.61	90.41 ± 6.79	*t* = -30.29	**<.001**
SBP (mmHg)	144.50 ± 16.89	141.45 ± 17.53	147.66 ± 15.60	*t* = -7.61	**<.001**
DBP (mmHg)	80.07 ± 9.98	79.29 ± 10.52	80.88 ± 9.33	*t* = -3.26	**0.001**
TG (mmol/L)	1.83 ± 1.20	1.54 ± 1.00	2.12 ± 1.31	*t* = -10.13	**<.001**
HDL-C (mmol/L)	1.26 ± 0.28	1.31 ± 0.29	1.20 ± 0.26	*t* = 8.19	**<.001**
FPG (mmol/L)	6.57 ± 1.69	6.33 ± 1.54	6.83 ± 1.80	*t* = -6.10	**<.001**

*t*, *t*-test; *Z*, Mann-Whitney U test; *χ*², Chi-square test; SD, standard deviation; M, Median; Q_1_, 1st Quartile; Q_3_, 3st Quartile.

Bold variables indicate a statistically significant difference between groups (MetS vs. Non-MetS) (*P*<0.05).

Regarding demographic characteristics, the mean age of the MetS group (69.43 ± 6.62 years) was significantly higher than that of the non-MetS group (68.40 ± 7.03 years) (*P* = 0.002). The distribution of age groups and the proportion of genders also showed statistically significant differences between the two groups (both *P* < 0.001). In terms of lifestyle, the mean BMI was 24.32 ± 3.00 kg/m², and the mean waist circumference was 85.32 ± 8.36 cm. The current smoking rate (12.95%) and current drinking rate (12.70%) in the MetS group were significantly lower than those in the non-MetS group (28.93% and 21.55%, respectively) (both *P* < 0.001). Regarding PA intensity, 79.89% of participants engaged in low-intensity PA, with only 20.11% achieving moderate or vigorous intensity. There were no statistically significant differences between the two groups in the distribution of PA levels, median weekly activity duration, or median total activity volume (all *P* > 0.05). For anthropometric and metabolic indicators, all showed statistically significant distribution differences between the two groups. The MetS group had significantly higher values compared to the non-MetS group for BMI (26.01 ± 2.57 kg/m² vs. 22.70 ± 2.44 kg/m²), waist circumference (90.41 ± 6.79 cm vs. 80.42 ± 6.61 cm), systolic blood pressure (SBP) (147.66 ± 15.60 mmHg vs. 141.45 ± 17.53 mmHg), diastolic blood pressure (DBP) (80.88 ± 9.33 mmHg vs. 79.29 ± 10.52 mmHg), triglycerides (TG) (2.12 ± 1.31 mmol/L vs. 1.54 ± 1.00 mmol/L), and fasting plasma glucose (FPG) (6.83 ± 1.80 mmol/L vs. 6.33 ± 1.54 mmol/L) (all P < 0.05). Conversely, HDL-C was significantly lower in the MetS group (1.20 ± 0.26 mmol/L vs. 1.31 ± 0.29 mmol/L, *P* < 0.001) (**Table 1**).

### 3.2 Multivariate logistic regression analysis

After adjusting for gender, age, BMI, smoking, and drinking, the results of the multivariate logistic regression analysis are shown in **Table 2**. Regarding MetS as a whole, compared to the low PA level, neither the moderate level (*OR* = 1.07, *95% CI*: 0.77-1.47, *P* = 0.694) nor the high level (*OR* = 0.55, *95% CI*: 0.09-3.35, *P* = 0.513) showed significant protective or risk associations. With each unit increase in weekly PA duration, the risk of MetS showed a slight increase (*OR* = 1.01, *95% CI*: 1.01-1.01, *P* = 0.025). Total PA volume was not significantly associated with MetS risk (*P* = 0.323). Among the MetS components, a significant and consistent association was found between PA and elevated TG. The moderate PA level was associated with a significantly higher risk of elevated TG compared to the low level (*OR* = 1.35, *95% CI*: 1.05-1.73, *P* = 0.021); each unit increase in weekly activity duration (*OR* = 1.01, *95% CI*: 1.01-1.01, *P* = 0.009) and total activity volume (*OR* = 1.00, *95% CI*: 1.00-1.00, *P* = 0.015) also corresponded to an increased risk of elevated TG. PA level, duration, and volume showed no statistically significant associations with the risk of central obesity, elevated BP, reduced HDL-C, or elevated FPG (all *P* > 0.05) (**Table 2**).

**Table 2:** Multivariate logistic regression analysis.

Outcome variables	PA level (ref: Low)				Activity time		Activity amount	
	Moderate vs. low		High vs. low					
	*OR* (*95%CI*)	*P*-value	*OR* (*95%CI*)	*P*-value	*OR* (*95%CI*)	*P*-value	*OR* (*95%CI*)	*P*-value
MetS	1.07 (0.77 ~ 1.47)	0.694	0.55 (0.09 ~ 3.35)	0.513	**1.01 (1.01 ~ 1.01)**	**0.025**	1.00 (1.00 ~ 1.00)	0.323
Central obesity	1.16 (0.82 ~ 1.65)	0.392	0.35 (0.05 ~ 2.60)	0.303	1.01 (1.00 ~ 1.01)	0.082	1.00 (1.00 ~ 1.00)	0.588
Elevated BP	1.08 (0.77 ~ 1.51)	0.663	0.85 (0.21 ~ 3.35)	0.814	1.00 (1.00 ~ 1.01)	0.779	1.00 (1.00 ~ 1.00)	0.841
**Elevated TG**	**1.35 (1.05 ~ 1.73)**	**0.021**	1.34 (0.40 ~ 4.51)	0.638	**1.01 (1.01 ~ 1.01)**	**0.009**	**1.01 (1.01 ~ 1.01)**	**0.015**
Reduced HDL-C	1.03 (0.79 ~ 1.34)	0.828	0.80 (0.20 ~ 3.24)	0.757	1.00 (1.00 ~ 1.01)	0.419	1.00 (1.00 ~ 1.00)	0.428
Elevated FPG	0.82 (0.61 ~ 1.10)	0.186	0.76 (0.20 ~ 2.92)	0.684	1.00 (0.99 ~ 1.00)	0.28	1.00 (1.00 ~ 1.00)	0.079

All models were adjusted for gender, age, BMI, smoking, and drinking.

Bolded OR and *P*-values indicate a statistically significant association between the PA indicator and the metabolic outcome (*P*<0.05).

### 3.3 Sensitivity analysis

To test the robustness of the main analysis results above, two sensitivity analyses were conducted. First, by combining the moderate and vigorous PA groups into one group and comparing it with the low-level group, the association with the risk of elevated TG remained statistically significant (*OR* = 1.28, *95% CI*: 1.01-1.63, *P* < 0.05), while associations with other metabolic outcomes were not statistically significant. Second, in the model without adjusting for BMI, the point estimate for the association between moderate PA level and elevated TG risk was 1.28 (*95% CI*: 1.00-1.63), and the association was not statistically significant (*P* = 0.051). The pattern of other results was largely consistent with the main analysis, indicating that the main findings have good robustness (**Table 3**).

**Table 3:** Sensitivity analyses for the association between physical activity and metabolic outcomes, *OR* (*95% CI*).

Analysis scenario	Main analysis (Reference)	Combined mod/high PA	Without BMI adjustment
	Moderate vs. low	Mod/high vs. low	Moderate vs. low
MetS	1.07 (0.77 ~ 1.47)	0.87 (0.68 ~ 1.11)	0.89 (0.70 ~ 1.14)
Central obesity	1.16 (0.82 ~ 1.65)	0.89 (0.70 ~ 1.14)	0.93 (0.72 ~ 1.18)
Elevated BP	1.08 (0.77 ~ 1.51)	1.05 (0.76 ~ 1.45)	1.08 (0.78 ~ 1.49)
**Elevated TG**	1.35 (1.05 ~ 1.73)*	1.28 (1.01 ~ 1.63)*	1.28 (1.00 ~ 1.63)
Reduced HDL-C	1.03 (0.79 ~ 1.34)	0.92 (0.72 ~ 1.18)	0.93 (0.73 ~ 1.20)
Elevated FPG	0.82 (0.61 ~ 1.10)	0.80 (0.60 ~ 1.07)	0.81 (0.60 ~ 1.09)

*P < 0.05. All models were adjusted for gender, age, smoking, and drinking. The main analysis was additionally adjusted for BMI.

Bolded ORs indicate that the association between PA and metabolic outcomes remained statistically significant after adjusting for different covariates, emphasizing the robustness of the findings.

### 3.4 Stratified analysis by body mass index and interaction tests

To further explore the potential modifying effect of BMI, stratified analyses were conducted according to BMI categories (underweight, normal weight, overweight, obese), and interactions were tested. For MetS as a whole, the association between PA level (moderate/high vs. low) and MetS did not differ significantly across BMI subgroups (*P* for interaction = 0.993). However, significant interactions were found for specific MetS components. In the analysis of elevated TG, the *P*-value for interaction was 0.006, indicating that the strength of the association between PA and elevated TG varied by BMI category. Specifically, among the normal-weight population, MVPA was significantly associated with elevated TG risk (*OR* = 1.71, *95% CI*: 1.25-2.35, *P* < 0.001); whereas in the underweight, overweight, and obese populations, this association was not statistically significant (all *P* > 0.05). For central obesity, elevated BP, reduced HDL-C, and elevated FPG, the associations between PA and these outcomes did not show significant heterogeneity across BMI subgroups (all *P* for interaction > 0.05) (**Table 4**).

**Table 4:** Subgroup analysis by BMI category and interaction tests.

Outcome variables	BMI category	Total, n (%)	Low PA group	Mod/high PA group	*OR* (*95% CI*)	*P-*value	*P* for interaction
MetS	underweight	38 (2.30)	0/30	0/8	1.00 (0.00 ~ Inf)*	1.000	0.993
	Normal Weight	971 (58.81)	237/766	62/205	1.04 (0.72 ~ 1.51)	0.830	
	Overweight	578 (35.01)	375/476	76/102	0.99 (0.58 ~ 1.67)	0.956	
	Obese	64 (3.88)	45/47	16/17	0.90 (0.07 ~ 11.90)	0.935	
Central obesity	underweight	38 (2.30)	0/30	0/8	1.00 (0.00 ~ Inf)*	1.000	0.785
	Normal Weight	971 (58.81)	278/766	71/205	0.99 (0.69 ~ 1.42)	0.966	
	Overweight	578 (35.01)	400/476	85/102	1.36 (0.73 ~ 2.53)	0.340	
	Obese	64 (3.88)	46/47	17/17	—*	0.998	
Elevated BP	underweight	38 (2.30)	19/30	4/8	0.71 (0.10 ~ 5.29)	0.741	0.331
	Normal Weight	971 (58.81)	598/766	168/205	1.27 (0.85 ~ 1.91)	0.25	
	Overweight	578 (35.01)	426/476	88/102	0.70 (0.37 ~ 1.34)	0.281	
	Obese	64 (3.88)	40/47	15/17	1.52 (0.25 ~ 9.31)	0.649	
**Elevated TG**	underweight	38 (2.30)	1/30	3/8	3.00 (0.08 ~ 107.45)	0.547	**0.006**
	Normal Weight	971 (58.81)	267/766	96/205	1.71 (1.25 ~ 2.35)	**<.001**	
	Overweight	578 (35.01)	230/476	45/102	0.86 (0.56 ~ 1.33)	0.498	
	Obese	64 (3.88)	23/47	7/17	0.64 (0.20 ~ 2.05)	0.450	
Reduced HDL-C	underweight	38 (2.30)	5/30	46061	0.61 (0.05 ~ 7.48)	0.702	0.664
	Normal Weight	971 (58.81)	269/766	2/8	1.12 (0.80 ~ 1.57)	0.514	
	Overweight	578 (35.01)	235/476	43/102	0.90 (0.57 ~ 1.42)	0.643	
	Obese	64 (3.88)	26/47	8/17	0.60 (0.19 ~ 1.94)	0.398	
Elevated FPG	underweight	38 (2.30)	21/30	6/8	3.81 (0.29 ~ 50.76)	0.311	0.851
	Normal Weight	971 (58.81)	594/766	154/205	0.87 (0.60 ~ 1.25)	0.444	
	Overweight	578 (35.01)	408/476	82/102	0.67 (0.39 ~ 1.18)	0.169	
	Obese	64 (3.88)	42/47	14/17	0.62 (0.12 ~ 3.09)	0.559	

Models were adjusted for gender, age, smoking, and drinking (BMI was not adjusted as the stratification was based on BMI). kg/m²).

*These estimates are subject to extreme uncertainty.

Bolded "P for Interaction" values (*P*=0.006) suggest that BMI significantly moderates this association, meaning the effect of PA on metabolic outcomes differs across BMI groups.

To further explore effect heterogeneity, we performed stratified analyses by gender and age. No significant interactions were found (all *P* for interaction > 0.05), suggesting that the metabolic effects of PA are relatively consistent across different genders and age groups in the elderly population.

## 4 Discussion

This study systematically analyzed the associations between PA levels and metabolic health indicators in a cohort of 1,651 community-dwelling older adults aged 60 years and above. The findings revealed a complex and somewhat paradoxical relationship between PA and MetS and its components. The main conclusions can be summarized as follows: First, at the overall level, conventionally defined MVPA did not demonstrate a protective effect against MetS or most of its components. Second, longer weekly PA duration was weakly positively associated with the risk of MetS, while moderate PA level, longer activity duration, and higher total activity volume were all significantly associated with an increased risk of elevated TG. Finally, the positive association between MVPA and elevated TG was particularly pronounced in individuals with normal body weight, suggesting that BMI plays a key role as an effect modifier. These findings challenge the simplistic notion that “more PA is always better,” indicating that the health benefits of PA in older adults require a more nuanced evaluation that considers activity patterns, intensity, and individual metabolic baseline.

Previous studies have confirmed that PA and exercise play a critical role in preventing MetS (39), with moderate or vigorous PA recommended for promoting health and preventing chronic diseases (36). The absence of a significant beneficial effect of MVPA on reducing MetS risk in this study contrasts with the conclusions of many studies advocating exercise for metabolic disease prevention (40, 41). This discrepancy may stem from the unique demographic and activity characteristics of the study population. The distribution of PA levels in this cohort was highly skewed, with an overall low level of PA among older adults—nearly 80% of participants were classified in the “low-level” group. This proportion is significantly higher than reported in other regions (42, 43), highlighting a significant public health issue of physical inactivity in this elderly population, which may be attributed to age-related functional decline, comorbidities, or cultural factors. However, this skewed distribution, particularly the extremely small number of participants in the high-intensity group (n=11), severely limited our statistical power to detect any meaningful protective or risk associations for this specific intensity level. The lack of a significant finding for the high-intensity group should therefore be interpreted with caution, as it may represent a Type II error. Additionally, the overall activity intensity in this population may be generally low. In this context, activities classified as “moderate” by the questionnaire may not have reached the threshold of energy expenditure and physiological stimulation required to effectively improve insulin sensitivity, promote lipid metabolism, and regulate blood pressure. Furthermore, the measured “activity duration” and “total activity volume” may have largely included low-intensity daily activities (e.g., walking, household chores), which are far less effective in improving cardiometabolic health than planned, threshold-meeting exercises (44). Thus, the null findings of this study may reflect the difficulty of significantly preventing MetS—a complex cluster of metabolic disorders—by merely increasing “activity quantity” without improving “activity quality” in an older population generally lacking effective intensity in their activities. This underscores the importance of reducing sedentary behavior and increasing PA in daily life for older adults. Interestingly, a recent study utilizing wearable accelerometer data from the UK Biobank (45) has shown a dose-response relationship between MVPA and reduced risk of metabolic syndrome and other chronic diseases in large cohorts of adults and older adults. The study also found that the metabolic benefits of physical activity are highly dependent on the type and context of the activity, suggesting that unstructured, low-intensity daily activities common among older adults may not provide the same cardiovascular and metabolic protection as structured exercise. This supports our hypothesis that the absence of observed benefits in our cohort may be due to a focus on low-intensity daily living activities rather than purposeful, threshold-level exercise.

Reduced PA has been confirmed to be associated with MetS-related factors such as obesity, hypertension, diabetes, and dyslipidemia. Regular PA can reduce body weight, improve blood pressure, and effectively influence lipid profiles related to metabolism. Studies show that for most people, moderate or lower-intensity exercise is sufficient to significantly improve lipid profiles, particularly low-density lipoprotein cholesterol and TG (46, 47). In contrast, this study found a positive association between PA and the risk of elevated TG in component analyses, which contradicts the findings of related studies (41). These results likely indicate the presence of “reverse causality,” meaning that PA is not the direct cause of MetS but rather that the diagnosis of the condition prompts older adults to pay greater attention to their health, leading them to actively increase or adopt PA. In other words, individuals may have consciously increased their PA, particularly the recorded “activity duration,” due to medical advice or self-health management driven by the detection of elevated TG or awareness of other metabolic risks (e.g., central obesity). This could explain the observed result in cross-sectional data that “individuals with elevated TG had longer activity durations.” The positive association between activity duration and overall MetS risk in the multivariate regression analysis (*OR* = 1.01) also provides indirect support for this explanation. Second, the potential for energy compensation after PA cannot be ruled out, although we acknowledge the lack of objective dietary data to directly support this hypothesis. Studies indicate that the direct effects of dietary patterns remain a primary risk factor for MetS. Older adults may unconsciously increase energy intake, particularly carbohydrates and fats, after PA, thereby fully or partially offsetting the energy expenditure from the activity, leading to unchanged or even increased TG levels (48). However, this remains a speculative mechanism that requires further investigation with concurrent dietary assessments in future studies. To prevent the rise in morbidity and mortality due to MetS, it is imperative to initiate large-scale community intervention programs focusing on lifestyle and dietary behaviors.

Among the components of MetS, obesity is the most common symptom, and its pathogenesis involves inflammation triggered by excessive adipose tissue accumulation (49). BMI, as a core indicator of overall obesity (50), may play a significant modifying role in the association between PA and MetS. The stratified analysis by BMI in this study profoundly reveals the critical role of BMI in the relationship between PA and metabolism. Sensitivity and subgroup analyses consistently indicate that BMI may not only confound this association but also serve as a significant effect modifier. In older adults with normal weight, the association between MVPA and elevated TG risk reached a strength of 1.71. This strongly suggests that “normal weight” is far from synonymous with “metabolic health.” This group may exhibit the “normal-weight metabolically obese” phenotype, characterized by a normal BMI but a high body fat percentage, insufficient muscle mass, and potentially excessive visceral fat area. Their underlying metabolic status (e.g., insulin resistance, chronic low-grade inflammation) may already be compromised (51). For them, conventional moderate-intensity PA may be insufficient to reverse their existing, underlying lipid metabolism disorders. Instead, due to the aforementioned reverse causality, this manifests statistically as a stronger risk association. This finding carries significant clinical implications: for older adults with normal weight, metabolic screening must not be overlooked. Their exercise prescriptions may require more refined design, emphasizing resistance training to increase muscle mass and improve body composition, thereby synergizing with aerobic exercise to optimize metabolic function.

This study, for the first time in a representative large-sample community-dwelling older adult population, revealed the important and complex phenomenon of the “PA-elevated TG” association modified by BMI, highlighting significant academic value and public health implications. However, several limitations exist: First, the cross-sectional design precludes any inference of causality. The observed positive associations, particularly between PA and elevated TG, may be subject to reverse causality—i.e., individuals diagnosed with or aware of their metabolic risk factors (like elevated TG) may have consciously increased their PA levels or duration as a health management strategy. Second, the use of self-reported PA via the IPAQ is susceptible to recall bias and social desirability bias, potentially leading to overestimation or underestimation of true activity levels, which could distort the true association. Third, the absence of objective body composition measurements and detailed dietary intake data limits the ability to control for these important confounding factors. Fourth, combining high and moderate PA groups for analysis, though done for statistical power considerations, may obscure the unique effects of truly vigorous-intensity activities.

## 5 Conclusion

This study indicates that in a community-dwelling older adult population, no expected inverse association was found between overall PA levels and MetS risk. Moderate-intensity PA was associated with higher TG levels, and this association was particularly evident in older adults with normal body weight. These findings should not be interpreted as physical activity being generally harmful for the elderly. Rather, they underscore the complexity of the PA-metabolism relationship and highlight the necessity of individualized assessments that consider a person’s baseline health status, body composition, and the specific type, intensity, and context of their physical activity when formulating public health guidelines and clinical recommendations. Future research should employ prospective cohort or intervention trial designs, incorporating objective PA measurement tools (e.g., accelerometers) and detailed dietary assessments, to clarify the causal relationship and biological mechanisms underlying this association.

## Data Availability

The original contributions presented in the study are included in the article/supplementary material. Further inquiries can be directed to the corresponding authors.
